# Effects of High Ambient Temperature on Small Intestinal Morphology and Colonic Microbiota in Weaned Piglets

**DOI:** 10.3390/ani12141743

**Published:** 2022-07-07

**Authors:** Shuaibing Xing, Shuai Chen, Ying Zhao, Yuheng Luo, Bing Yu, Jun He, Zhiqing Huang, Ping Zheng, Xiangbing Mao, Junqiu Luo, Hui Yan, Jie Yu

**Affiliations:** 1 Key Laboratory for Animal Disease-Resistance Nutrition of Sichuan Province, Animal Nutrition Institute, Sichuan Agricultural University, Chengdu 611130, China; xsb19951120@163.com (S.X.); dwchen@sicau.edu.cn (S.C.); luoluo212@126.com (Y.L.); ybingtian@163.com (B.Y.); hejun8067@163.com (J.H.); zqhuang@sicau.edu.cn (Z.H.); zpind05@163.com (P.Z.); acatmxb2003@163.com (X.M.); 13910@sicau.edu.cn (J.L.); yan.hui@sicau.edu.cn (H.Y.); 2 Sichuan Tequ Agriculture and Animal Husbandry Technology Group Co., Ltd., Chengdu 610207, China; yingzhao820@126.com

**Keywords:** weaned piglets, high ambient temperature, growth performance, small intestinal morphology, colonic microbiota

## Abstract

**Simple Summary:**

Thermal stress (TS) is a critical challenge in the swine industry, but knowledge about the influence of TS on intestinal development and microbiota in piglets is still scarce. Thus, the present study investigated the effects of high ambient temperature on growth performance, small intestinal morphology, and colonic microbiota in weaned piglets. Our results showed that high ambient temperature had negative effects on growth performance and colonic microbiota of weaned piglets.

**Abstract:**

A total of 16 crossbred (Duroc × Landrace × Yorkshire) barrows, with an average initial body weight of 8.61 ± 0.24 kg (28 days of age), were randomly allotted into the control group (CON group) and high ambient temperature group (HT group) with 8 replicates per group, 1 pig per replicate. The ambient temperature of the CON group was controlled at 26 ± 1 °C, and the HT group was controlled at 35 ± 1 °C. The study lasted for 21 days. Our results showed that high ambient temperature significantly decreased the average daily feed intake (ADFI) and average daily gain (ADG) of piglets (*p* < 0.05), and the feed-to-gain ratio was significantly increased (*p* < 0.05). The liver index, spleen index, and thymus index of piglets in the HT group were significantly decreased (*p* < 0.05). The villous height (VH) of the duodenum, jejunum, and ileum of piglets in the HT group was significantly decreased (*p* < 0.05), whereas the crypt depth (CD) of the duodenum was significantly increased (*p* < 0.05), and the VH-to-CD ratio of the duodenum and ileum was significantly decreased (*p* < 0.05). The piglets in the HT group showed a higher (*p* < 0.05) observed-species index, PD whole tree index, and Shannon index, indicating that there was a significant difference in species richness and diversity between the two groups. At the genus level, the piglets in the HT group showed a greater (*p* < 0.05) percent of *Desulfovibrio*, *Occillibater*, and *Catenisphaera*. HT reduced glycan biosynthesis and metabolism, transport and catabolism, lipid metabolism, amino acids metabolism, secondary metabolites biosynthesis, aging, endocrine system, signaling molecules, and interaction of colon microbiota (*p* < 0.05), and increased signal transduction, cell motility, transcription, and genetic information processing (*p* < 0.05).

## 1. Introduction

In tropical regions and in temperate countries, summer high temperature causes huge economic losses in the pig industry [1]. These losses are related to the negative effects of heat stress (HS) on reproductive and growth performances [2]. Several research reports indicate that intestinal microbiota can help the host to digest and absorb, providing nutrients and enzymes to maintain the normal physiological functions of the body [3,4]. Tarini et al. [5] showed that cecal microbes can produce a large number of short-chain fatty acids to provide energy for the host through fermentation. In addition, intestinal microbiota also constitutes the intestinal microbial barrier, impacting intestinal morphology, feed digestion, nutrient absorption, and intestinal immune function. Moreover, the structure and composition of the intestinal microbiota are affected by heredity, diet, age, drug, disease, and environment [6,7,8,9].

It is well known that HS has detrimental effects on pig’s growth performance and health, high temperature in summer is the main cause of HS in animals [10,11]. Furthermore, HS destroys the tissue structure of intestinal epithelium and leads to an increase in intestinal permeability, and the susceptibility of animals to pathogenic bacteria increases. HS causes a change in the type and function of microbiota, changes the level of absorption rate, and affects the overall gut health, which may be the first step in the development of malnutrition and intestinal inflammation in animals [12,13,14]. However, it is well recognized that many of these bacteria have not yet been cultured in the laboratory. Recent advances in high-throughput sequencing technologies, coupled with efficient bioinformatics tools, have enabled in-depth analysis of the intestinal microbiota community of weaned piglets. In contrast to traditional microbiological techniques, these approaches provide a relatively comprehensive description of the intestinal microbiota. In the current study, we sought to analyze the effect of high temperature on community structure and composition of colon microbiota in weaned piglets and evaluate how these changes in microbiota composition interplay with animal performance.

## 2. Materials and Methods

### 2.1. Ethical Approval

All animal procedures were performed according to protocols approved by the Institutional Animal Care and Use Committee of Sichuan Agricultural University (20190053).

### 2.2. Animals, Diets and Experimental Design

The experiment was conducted in the teaching and scientific research base of the Animal Nutrition Research Institute of Sichuan Agricultural University. A total of 16 crossbred (Duroc × Landrace × Yorkshire) barrows, with an average initial body weight of 8.61 ± 0.24 kg (28 days of age), were randomly allotted into the control group (CON group) and high ambient temperature group (HT group) with 8 replicates per group, 1 pig per replicate. All pigs were fed in piglet metabolic cages (1 piglet per cage). The ambient temperature of the CON group was maintained at 26 ± 1 °C, the HT group was maintained at 35 ± 1 °C, and all pigs were fed the basal diet. The ambient temperature was maintained by adjusting the number of warming lights on, the humidity was controlled at 65–80%. The study lasted for 21 days. The nutrient levels in the feed met NRC (2012) 7–11 kg stage requirements (Table 1). All pigs were handfed four times/d (8:00, 12:00, 16:00, and 20:00 h) in groove feeders, and free to gather drinking water. Feed intake was recorded daily. All pigs were individually weighed at the start and the end of the trial to calculate average daily body weight gain (ADG), average daily feed intake (ADFI), and the feed to gain ratio (F/G). All rooms were cleaned regularly and disinfected alternately to maintain sanitation.

### 2.3. Samples Collection

At the end of the trial, the trough was emptied and all pigs were fasted for 12 h. On the 22nd day of the experiment, all piglets were weighed and recorded, and then all piglets were fed for the last time. After two hours, all piglets were slaughtered in order. All piglets were killed with sodium pentobarbital (200 mg kg ^−1^ BW). After dissection, the heart, liver (gallbladder removed), spleen, lung, kidney, and thymus were taken out, weighed, and recorded. The gastrointestinal tract was separated, and the tissues of the duodenum (1 cm), jejunum (3 cm), and ileum (2 cm) were collected and washed with 0.9% stroke-physiological saline solution, then were put into 4% paraformaldehyde (PFA) solution for fixation. Approximately 4 g of digesta from the middle colon of each piglet was transferred into a sterile 2 mL tube and immediately frozen at −80 °C for the analysis of community structure and composition of colonic microbiota.

### 2.4. Organ Index Calculation Formula

organ index = organ weight (g)/body weight (kg)

### 2.5. Intestinal Morphology and Structure

Histological analyses were performed as previously described [15]. Briefly, the gastrointestinal tract was separated, and the tissues of the duodenum (1 cm), jejunum (3 cm), and ileum (2 cm) were collected and washed with 0.9% stroke-physiological saline solution, then put into 4% paraformaldehyde (PFA) solution for 24 h. The fixed intestinal samples were dehydrated in ethanol, cleared in xylene, and embedded in paraffin wax. Then, the samples were transverse sectioned at a 4 μm thickness and installed on glass slides. Paraffin sections were dewaxed to water with xylene, ethanol, and distilled water, and stained by hematoxylin-eosin staining (H&E). Finally, the sections were sealed with neutral gum after being dehydrated again for a light microscopy examination. At least three 40× visual fields were randomly selected for each section in each group. When taking photos, we tried to fill the whole field of vision with the organization to ensure that the background light of each photo is consistent. Five intact villi were selected from each section, and Image Pro Plus 6.0 software was used to measure the villous height (mm), the crypt depth (mm), and the villous height-to-crypt depth ratio.

### 2.6. DNA Extraction and 16S rRNA Gene Sequencing

According to the manufacturer’s instructions, microbiota DNA from colonic samples was extracted using the Omega E.Z.N.A.TM Stool DNA Isolation kit (Omega Bio-Tek, Norcross, GA, USA). The quality of DNA was determined by 1% agarose gel electrophoresis. The concentration and purity of DNA were examined by a NanoDropTM spectrophotometer (Thermo Fisher Scientific Inc, Waltham, MA, USA). The V3-V4 high-variation region of the 16S rRNA [16,17,18] gene in bacterial DNA was amplified by using the primer pairs 338F (5′-ACTCCTACGGGAGGCAGCAG-3′) and 806R (5′-GGACTACHVGGGTWTCTAAT-3′). All PCR reactions were performed in 30 µL solutions containing 15 µL Phusion^®^ High-Fidelity PCR Master Mix (New England Biolabs), 0.2 µM forward and reverse primers, and about 10 ng template DNA. The PCR amplification program was as follows: initial denaturation at 98 °C for 1 min, 30 cycles of denaturation at 98 °C for 10 s, annealing at 50 °C for 30 s, elongation at 72 °C for 30 s, and a final extension step at 72 °C for 5 min. The resulting PCR products were detected using 2% agarose gels, further purified using the GeneJETTM Gel Extraction Kit (Thermo Fisher Scientific Inc., Waltham, MA, USA), and quantified using a Qubit 2.0 fluorometer (Thermo Fisher Scientific Inc., Waltham, MA, USA) according to the manufacturer’s protocols. Purified amplicons were pooled in equimolar amounts and subjected to single-end sequencing on the lonS5TMXL sequencing platform conducted by Novogene company (Beijing, China).

### 2.7. Bioinformatics Analysis for Colonic Microbiota

The raw reads were quality filtered by using Cutadapt software (V1.9.1, http://cutadapt.readthedocs.io/en/stable/, accessed on 13 November 2019). All clean reads were aligned with the reference database (Silva database, https://www.arb-silva.de/, accessed on 13 November 2019) using the UCHIME algorithm (UCHIME Algorithm, http://www.drive5.com/usearch/manual/uchime_algo.html, accessed on 13 November 2019). Operational taxonomic units (OTUs) were clustered with a 97% similarity. According to OTUs clustering results, on the one hand, we annotated each out sequence to obtain the corresponding species information and species abundance distribution. At the same time, OTUs abundance, alpha diversity calculation, Venn diagram, and other analyses were carried out to obtain species richness, evenness information, and common and unique OTUs information among different samples or groups within the sample. On the other hand, OTUs can be compared with multiple sequences and a phylogenetic tree can be constructed to explore the differences of community structure among different samples or groups through PCoA dimension reduction analysis. *t*-test, Anosim, and MRPP were used to test the significance of species composition and community structure. The annotated results of the amplified products were associated with the corresponding functional database, and the function prediction analysis of microbiota communities in ecological samples was carried out by using the Tax4fun software.

### 2.8. Statistical Analysis

Data on growth performance were obtained with each metabolic cage as an experimental unit. Furthermore, a *t*-test of SPSS 20.0 was used to analyze the experimental data, and the results were shown as the mean values ± SEM. Data for the bacterial community were analyzed with all colonic digesta samples in each group. Standardized OTUs reads were applied to analyze bacterial diversity by the guidance of R software. The abundance of bacteria at the phylum and genus levels was shown as bar plots. The comparative analysis between the two groups was conducted by using the method of Wilcox rank-sum test and *t*-test. It was considered a significant difference if *p* < 0.05.

## 3. Results

### 3.1. Growth Performance

As shown in Table 2, compared with the CON group, the ADFI and ADG of piglets in the HT group were decreased by 43.13% and 50.36% (*p* < 0.05), respectively, and the feed-to-gain ratio was increased by 16.06% (*p* < 0.05).

### 3.2. Organs Index

As shown in Table 3, compared with the CON group, the liver index, spleen index, and thymus index of piglets in the HT group were decreased (*p* < 0.05), but there was no difference in the heart index, lung index, and kidney index (*p* > 0.05).

### 3.3. Intestinal Morphology

**Figure 1:**
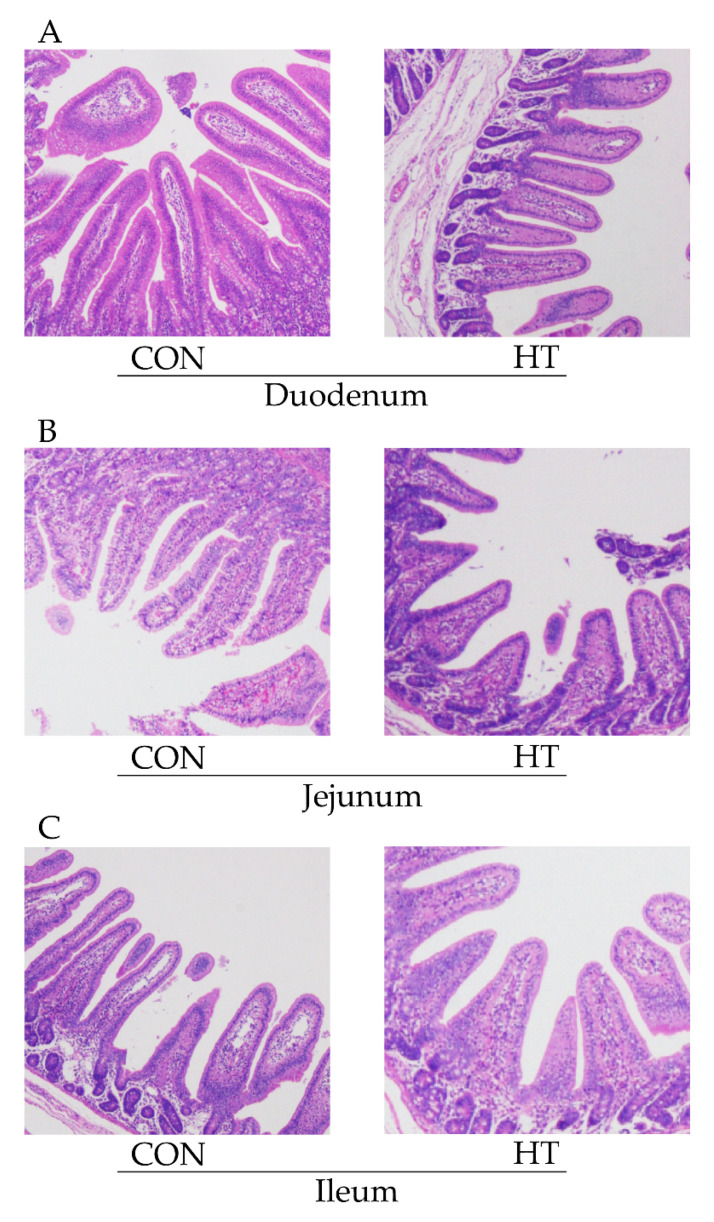
Effects of high ambient temperature on duodenum morphology (**A**), jejunum morphology (**B**), and ileum morphology (**C**) in wean piglets (scale bar: 500 μm). CON group, room temperature was controlled at 26 ± 1 °C; HT group, room temperature was controlled at 35 ± 1 °C.

The H&E staining results of duodenum morphology (A), jejunum morphology (B), and ileum morphology (C) in weaned piglets are shown in Figure 1. As shown in Table 4, compared with the CON group, the villous height (VH) of the duodenum, jejunum, and ileum of piglets in the HT group were decreased *(p* < 0.05), the crypt depth (CD) of the duodenum was increased (*p* < 0.05), and the VH-to-CD ratio of the duodenum and ileum were decreased (*p* < 0.05).

### 3.4. Alpha Diversity

**Figure 2:**
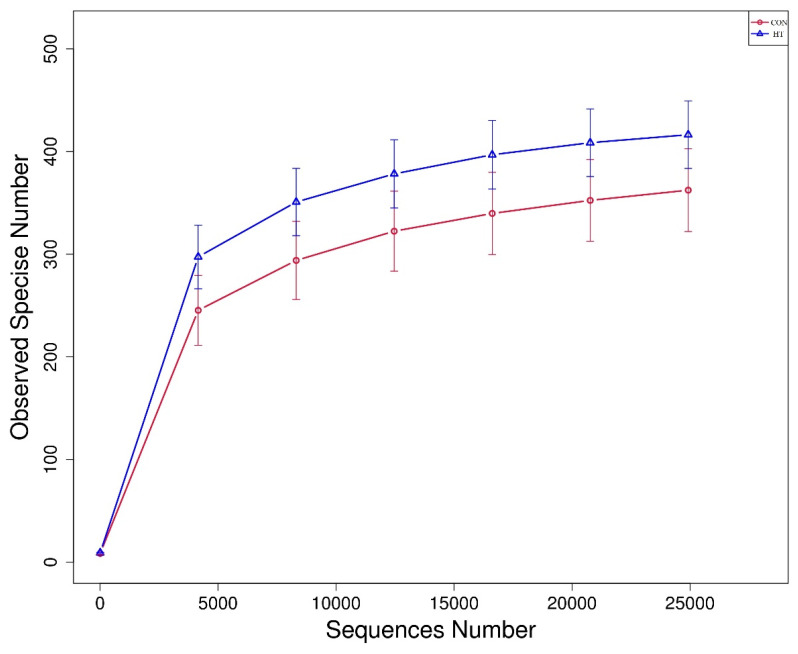
Rarefaction curve. CON group, room temperature was controlled at 26 ± 1 °C; HT group, room temperature was controlled at 35 ± 1 °C.

**Figure 3:**
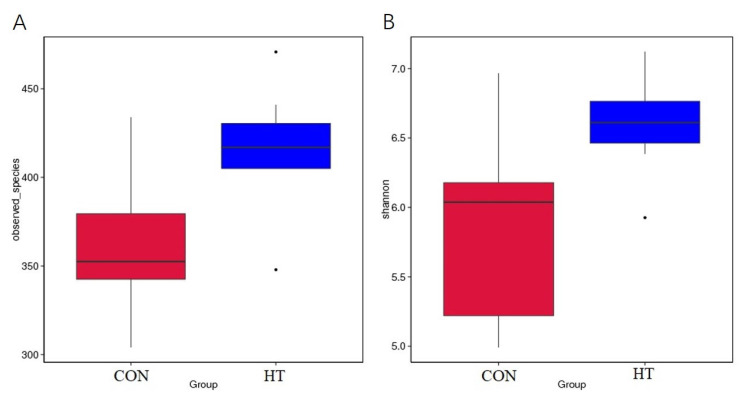
Analysis of differences between groups by observed species boxplot (**A**) and Shannon boxplot (**B**). CON group, room temperature was controlled at 26 ± 1 °C; HT group, room temperature was controlled at 35 ± 1 °C.

A total of 16 colonic content samples of piglets were divided into the CON group and HT group. After the original deplaning data were obtained from loS5TMXL, averages of 84,926 reads were measured per sample. A barcode sequence was used to distinguish the data of each sample, and 80,415 valid data for subsequent analysis were obtained after chimeric filtering, with the quality control efficiency reaching 94.83%. The rarefaction curve [19] mainly reflects the rationality of sequencing data volume and indirectly the richness of species in samples, as shown in Figure 2, where the curve tends to be flat, indicating that the sequencing data amount is reasonable. The alpha diversity was used to evaluate the differences in species richness and diversity of microbiota communities in each sample [20]. As shown in Table 5 and Figure 3, compared with the CON group, the observed-species index and Shannon index in the HT group were significantly increased (*p* < 0.05), indicating that the species richness and diversity of colonic microbiota in the HT group were increased.

### 3.5. Beta Diversity

**Figure 4:**
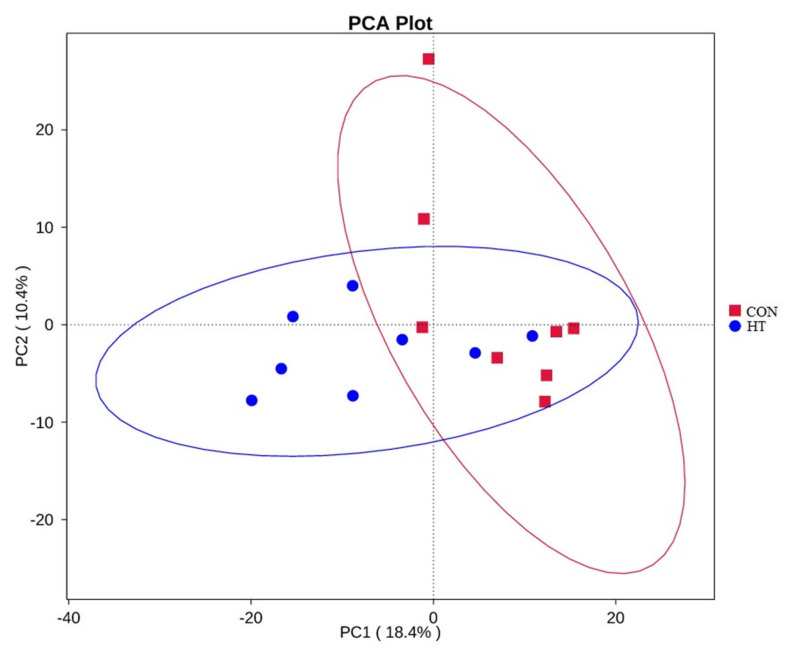
Principal component analysis (PCA) based on OTU level. CON group, room temperature was controlled at 26 ± 1 °C; HT group, room temperature was controlled at 35 ± 1 °C.

The principal component analysis (PCA) [21] based on the distance of all individual samples accounted for by Unifrac is shown in Figure 4. It can be seen from Figure 4 that the HT group formed a cluster different from that of the CON group. Anosim analysis [22] is mainly used to test whether the difference between groups is significantly greater than that within groups, and to judge whether grouping is reasonable. As can be seen from Table 6, anosim analysis results are R > 0, *p* < 0.05, indicating that the difference between groups is significant, and the statistical results are reliable.

### 3.6. Colonic Bacterial Community

**Figure 5:**
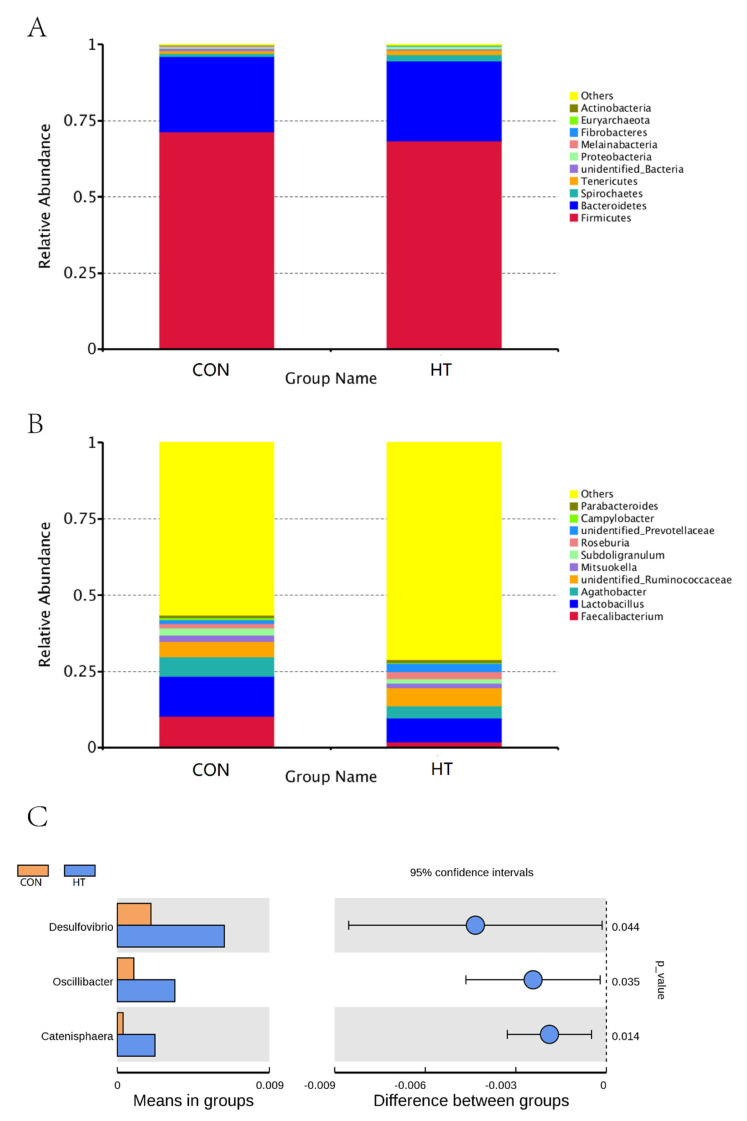
Display of species relative abundance and analysis of species differences between groups. Relative abundance of species on phylum level (**A**); relative abundance of species on genus level (**B**); differences species at genus level (**C**); CON group, room temperature was controlled at 26 ± 1 °C; HT group, room temperature was controlled at 35 ± 1 °C.

The OTUs sequences were annotated with the silva 132 database, and the ratio of annotation to phylum level was 98.52%, class level 96.16%, order level 91.14%, family level 87.15%, genus level 37.22%, and species level 13.88%. Figure 5A,B shows the main dominant species of colonic microbiota community at the phylum level and genus level in each group. At the phylum level, the main dominant species and abundance ratio of colonic microbiota in the control group were *Firmicutes* (71.45%), *Bacteroidetes* (24.73%), and *Spirochaetes* (1.04%), respectively. The high-temperature group was *Firmicutes* (68.44%), *Bacteroidetes* (26.17%), and *Spirochaetes* (2.06%, Figure 5A). As shown in Figure 5C, compared with the CON group, the percent of *Desulfovibrio*, *Oscillibacter*, and *Catenisphaera* in the HT group was increased (*p* < 0.05).

### 3.7. Predicted Functional Profiles of Microbiota Communities Using Tax4Fun

**Figure 6:**
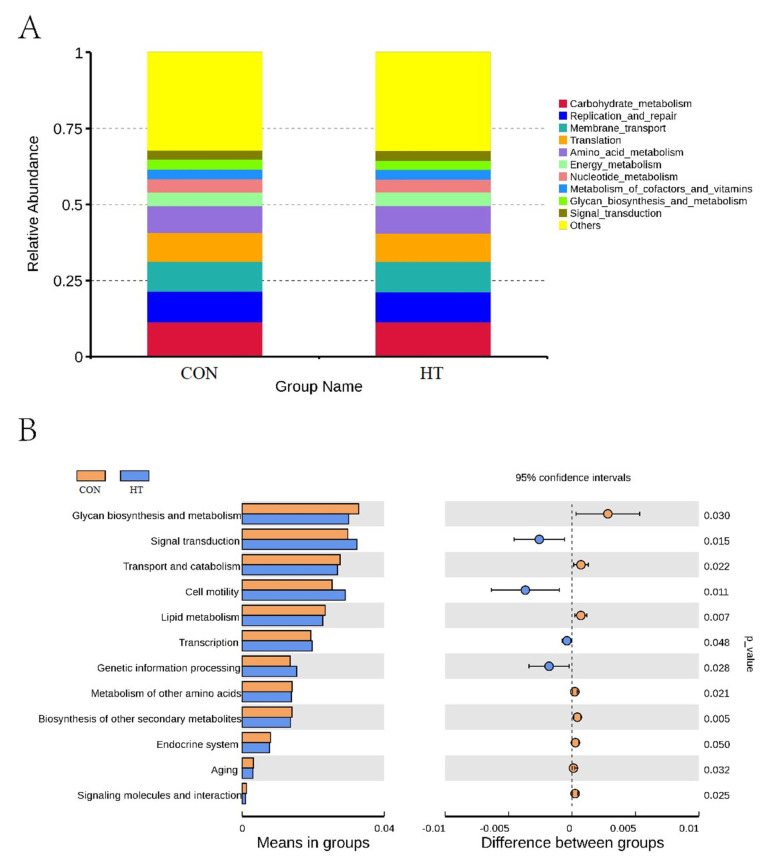
Function prediction. (**A**), Tax4fun function annotation relative abundance histogram; (**B**), functional differences of intestinal flora; CON group, room temperature was controlled at 26 ± 1 °C; HT group, room temperature was controlled at 35 ± 1 °C.

The metabolic function of colonic microbiota was obtained by using Tax4Fun function prediction [23]. As shown in Figure 6A, the top 10 functional units of colonic flora are carbohydrate metabolism, replication and repair, membrane transport, translation, amino acid metabolism, energy metabolism, nucleotide metabolism, metabolism of cofactors and vitamins, glycan biosynthesis and metabolism, and signal transduction. *t*-test difference analysis was carried out based on function annotation results. Figure 6B shows that compared with CON, HT reduced glycan biosynthesis and metabolism, transport and catabolism, lipid metabolism, amino acids metabolism, secondary metabolites biosynthesis, aging, endocrine system, signaling molecules, and interaction of colon microbiota (*p* < 0.05), and increased signal transduction, cell motility, transcription, and genetic information processing (*p* < 0.05).

## 4. Discussion

Pigs lack functional sweat glands and have a thick subcutaneous fat layer, so they have poor heat dissipation ability. When pigs are under HS, they mainly reduce their metabolic heat consumption by reducing feed intake to adapt to high environmental temperatures [24]. The results of our experiment showed that the ADFI of weaned piglets in the high-temperature group significantly decreased by 43.13%, which was an effective adjustment measure made by piglets to adapt to HS. In addition, the decrease in ADG and the increase in the feed-to-gain ratio indicated that HS led to the decrease in growth performance and economic benefit of weaned piglets. The results of Mayorga [25] and Oliveira [26] on fattening pigs showed that HS significantly reduced ADFI and ADG, which was basically consistent with the results of this experiment.

Immune organs consist of the central immune organs (such as the thymus) and peripheral immune organs (such as the spleen) [27]. The status of immune organs can determine immune function, but the preliminary indicators of immune organs are measured by the immune organ index and immune organ weight [28]. During heat stress, animals redistribute blood to the peripheral body surface, resulting in vasoconstriction, blood flow, and nutrient reduction in the gastrointestinal tract [29]. This will inhibit the development of internal organs. In our study, high temperature had no effect on heart index, lung index, and kidney index, but the liver index, spleen index, and thymus index of piglets in the HT group were significantly decreased. By this token detail, HS reduces immune function by suppressing the development of immune organs.

The most important characteristic of intestinal epithelial morphogenesis is the repetitive compartmentalized structures of crypt-villus units, which are crucial for maintaining intestinal homeostasis and functions [30]. Intestinal ischemia and hypoxia can inhibit the growth and development of intestinal epithelial cells, which will destroy the integrity of intestinal morphology [31]. An intact morphological structure of the intestine is important for nutrient utilization and absorption and is associated with longer villi which is a good indicator of a healthy gut; thus, its reduction and damage can be cautiously used to represent increasing intestinal permeability and infiltration of endotoxins [32]. In this experiment, heat stress significantly reduced the villi height and the villi-to-crypts ratio in the duodenum and ileum, which is consistent with the results of Yu [33] and Liu [34], this indicates that HS causes intestinal damage mainly in the duodenum and ileum.

The activity of the microbiota that settles in an animal’s gut affects many aspects of animal health. In the healthy state of animals, they can ferment non-digestible substances to provide nutrients and energy for the host and maintain a balance with the host’s metabolism and immune system [35,36]. However, the negative effects are that they may also act as inflammation and infection sources, inducing problems such as gastrointestinal diseases, inflammation, and obesity [37]. As described by Rojo [38], the decrease in the abundance of *Lactobacillus* under HS may be the main reason for the imbalance of intestinal flora and the occurrence of intestinal inflammation. The main active node of monogastric mammalian microbiota is the colon. Therefore, the collected colon contents were selected as the research object to explore the influence of HS on the microbiota community structure composition of weaned piglets. In this experiment, the colon-species index, PD-whole-tree index, and Shannon index of weaned piglets were significantly increased under the condition of HS, which indicated that HS could increase the richness and diversity of intestinal microbiota community of weaned piglets and change the composition of intestinal microbiota community. Through the species analysis of gene-level difference, we found that HS leads to a significant increase in the abundance ratio of *Desulfovibrio*, *Oscillibacter*, and *Catenisphaera* in the colon flora of weaned piglets. Sulfurized Vibrio belongs to sulphate-reducing bacteria (SRB), which can produce hydrogen sulfide with sulphate metabolism and damage the intestinal epithelial cells. Rowan [39] showed that in ulcerative colitis, the positive rate of *Desulfovibrio* was significantly increased and the increase in *Desulfovibrio* also represented the change of colonic microbiota structure in patients with ulcerative colitis. In this experiment, the abundance of *Desulfovibrio* in the colon flora of weaned piglets increased after HS, revealing that HS may induce ulcerative colitis in weaned piglets. Lam [40] showed that *Oscillibacter* was an important intestinal microbiota mediating a high-fat diet to induce intestinal microbial barrier dysfunction, and there was a negative correlation between intestinal microbial barrier function and *Oscillibacter* content. In the mouse model of ulcerative colitis established by Wang et al. [41], *Oscillibacter* with a higher level of fecal microbiota was observed. Thus, *Oscillibacter* may be directly involved in regulating the integrity of the intestinal barrier, but the exact mechanism remains unclear. *Oscillibacter* significantly increased in the colon of weaned piglets under the condition of HS, indicating that *Oscillibacter* may mediate HS to break the integrity of the intestinal morphological structure and reduce intestinal barrier function. The specific mechanism remains to be further studied. *Catenisphaera* belongs to *Erysipelotrichaceae* [42,43] and is closely related to host metabolism and inflammatory diseases. HS leads to a significant increase in *Catenisphaera* in the colon, which may induce inflammation in piglets and reduce metabolic functions of the body. From the functional prediction results, the core colonic flora of weaned piglets is mainly involved in carbohydrate metabolism, replication and repair, transmembrane transport, and translation. HS causes changes in the function of the core flora of the colon. The reduction in metabolism-related functions may affect growth performance, while other functional changes may help the host to adapt to HS.

In a word, in order to adapt to HS, weaned piglets were found to reduce feed intake. In addition, the disorder of colon flora structure and function may be one of the main factors affecting the growth performance and health of piglets under HS.

## 5. Conclusions

In summary, a duration of 3 weeks of high ambient temperature can cause a reduction in growth performance, suppress the development of immune organs, and affect the composition of the colonic microbiota community in weaned piglets. The inhibition of growth performance might be associated with colonic microbiota dysfunction induced by abnormal metabolism and nutrient absorption in weaned piglets.

## Figures and Tables

**Table 1:** Composition and nutrients levels of the basal diet (air-dry basis, %).

Items	%	Nutrients Levels ^3^	%
Corn	25.00	DE, Mcal/kg	14.82
Extruded corn	30.50	CP	18.35
Peeling soybean meal	10.00	Ca	0.62
Extruded soybean	7.00	TP	0.55
Wheat bran	1.45	AP	0.36
Rice, broken	7.90	*D*-Lys	1.35
Soy protein concentrate	3.50	*D*-Met	0.42
Plasma protein powder	2.00	*D*-Trp	0.24
Fish meal	3.50	*D*-Thr	0.79
Whey powder	3.00		
Soybean oil	2.00		
Limestone	0.57		
CaHPO_4_	0.38		
Salt	0.30		
*L*-Lys•HCl	0.39		
*DL*-Met	0.12		
*L*-Thr	0.04		
Chloride choline	0.10		
Vitamin premix ^1^	0.05		
Mineral premix ^2^	0.20		
Sucrose	2.00		
Total	100.00		

^1^ The vitamin premix provides the following per kilogram of diet: VA 8000 IU, VD_3_ 2000 IU, VE 25.0 IU, VK_3_ 1.2 mg, VB_1_ 2.5 mg, VB_2_ 6.5 mg, VB_6_ 10.0 mg, VB_12_ 50.0 μg, biotin 0.15 mg, folic acid 1.0 mg, *D*-pantothenic acid 20.0 mg, nicotinic acid 45.0 mg. ^2^ The mineral premix provides the following per kilogram of diet: Fe(FeSO_4_•H_2_O) 100 mg, Zn(ZnSO_4_·H_2_O) 100 mg, Mn(MnSO_4_·H_2_O) 4 mg, Se(Na_2_SeO_3_) 0.35 mg, Cu(CuSO_4_·5H_2_O) 100 mg, I(KI) 0.3 mg. ^3^ Nutrient levels were calculated values.

**Table 2:** Effects of high temperature on growth performance in weaned piglets.

Items	CON Group	HT Group	*p*-Value
Initial weight, kg	8.61 ± 0.24	8.61 ± 0.24	0.971
Final weight, kg	17.20 ± 0.72 ^a^	12.86 ± 0.31 ^b^	0.001
ADFI, g/d	559.20 ± 37.42 ^a^	318.03 ± 17.05 ^b^	0.001
ADG, g/d	408.93 ± 25.51 ^a^	202.98 ± 13.00 ^b^	0.001
F/G	1.37 ± 0.02 ^a^	1.59 ± 0.08 ^b^	0.022

Results are presented as the mean ± S.E.M. *n* = 8. CON group, ambient temperature was controlled at 26 ± 1 °C; HT group, ambient temperature was controlled at 35 ± 1 °C; ADFI, average daily feed intake; ADG, average daily gain; F/G, feed to gain ratio. ^a,b^ Superscripts indicated differences among different groups.

**Table 3:** Effects of high temperature on organs index in weaned piglets.

Organ Index	CON Group	HT Group	*p*-Value
Heart	5.05 ± 0.17	4.88 ± 0.14	0.467
Liver	27.28 ± 0.67 ^a^	23.41 ± 1.02 ^b^	0.007
Spleen	2.31 ± 0.23 ^a^	1.72 ± 0.08 ^b^	0.030
Lung	9.95 ± 0.55	8.87 ± 0.36	0.128
Kidney	5.58 ± 0.20	5.12 ± 0.25	0.167
Thymus	1.43 ± 0.10 ^a^	0.93 ± 0.08 ^b^	0.001

Results are presented as the mean ± S.E.M. *n* = 8. CON group, room temperature was controlled at 26 ± 1 °C; HT group, room temperature was controlled at 35 ± 1 °C. ^a,b^ Superscripts indicated differences among different groups.

**Table 4:** Effects of high temperature on intestinal morphology in weaned piglets.

Items	CON Group	HT Group	*p*-Value
Duodenum			
Villous height, μm	511.94 ± 28.20 ^a^	421.02 ± 21.54 ^b^	0.023
Crypt depth, μm	162.44 ± 11.50 ^a^	216.64 ± 22.18 ^b^	0.048
VH/CD	3.25 ± 0.27 ^a^	2.09 ± 0.23 ^b^	0.005
Jejunum			
Villous height, μm	366.19 ± 19.22 ^a^	281.48 ± 19.11 ^b^	0.007
Crypt depth, μm	128.49 ± 15.65	137.20 ± 13.54	0.681
VH/CD	3.27 ± 0.52	2.18 ± 0.25	0.080
Ileum			
Villous height, μm	389.26 ± 26.44 ^a^	291.34 ± 21.91 ^b^	0.013
Crypt depth, μm	134.11 ± 12.05	144.03 ± 12.51	0.577
VH/CD	3.13 ± 0.42 ^a^	2.08 ± 0.17 ^b^	0.037

Results are presented as the mean ± S.E.M. *n* = 8. CON group, room temperature was controlled at 26 ± 1 °C; HT group, room temperature was controlled at 35 ± 1 °C; VH/CD, the villous height-to-crypt depth ratio. ^a,b^ Superscripts indicated differences among different groups.

**Table 5:** Alpha diversity indexes.

Items	CON Group	HT Group	*t*-Test	Rank Sum Test
Observed-species index	362	416	0.016	0.045
Shannon index	5.855	6.595	0.019	0.028

Results are presented as mean ± S.E.M. *n* = 8. CON group, room temperature was controlled at 26 ± 1 °C; HT group, room temperature was controlled at 35 ± 1 °C.

**Table 6:** Anosim analysis.

Groups	R-Value	*p*-Value
C-T	0.3259	0.015

Results are presented as the mean ± S.E.M. *n* = 8. CON group, room temperature was controlled at 26 ± 1 °C; HT group, room temperature was controlled at 35 ± 1 °C; The R-value is between (−1, 1), and R-value > 0 indicates significant difference between groups. The reliability of statistical analysis can be represented by *p*-value and *p* < 0.05 indicates statistical significance.

## Data Availability

The data are available on request from the corresponding author.
